# Supplementary material for: A Fluorescence-Based Method to Measure ADP/ATP Exchange of Recombinant Adenine Nucleotide Translocase in Liposomes
Source: Biomolecules. 2020 Apr 29;10(5):685. doi: 10.3390/biom10050685 (PMC7277544; doi:10.3390/biom10050685)
Supplement: Supplementary file 1 [file biomolecules-10-00685-s001.pdf]

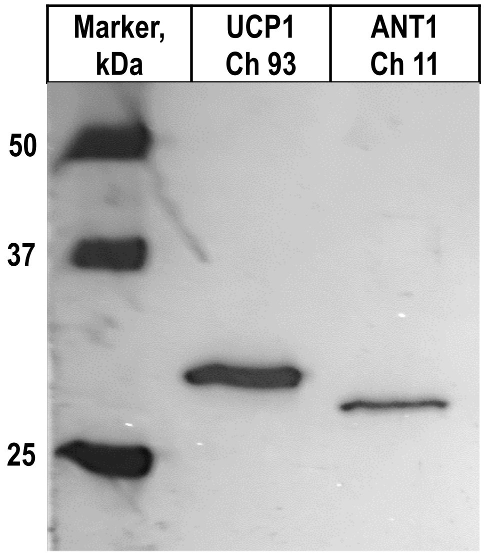

Supplementary Fig. 1 Silverstaining of recombinant murine uncoupling protein 1 (mUCP1) and murine adenine nucleotide translocase 1 (mANT1) reconstituted into liposomes.

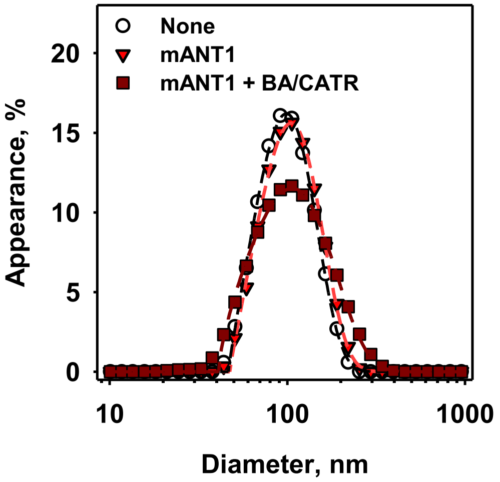

Supplementary Fig. 2 Sizes of liposomes after extrusion through 100 nm filters. In all measurements, the buffer contained 50 mM Na_2SO_4, 10 mM Tris, 10 mM MES, and 0.6 mM EGTA at pH = 7.34 and T = 297 K. Lipid membranes were made of 45:45:10 mol% of DOPC, DOPE, and CL, respectively. The lipid concentration was 1.0 mg/mL and liposome diameter was approximately 100 nm. The concentration of ANT1 was 8.67 µg/mL, and the concentration of BA and CATR was 100 µM.

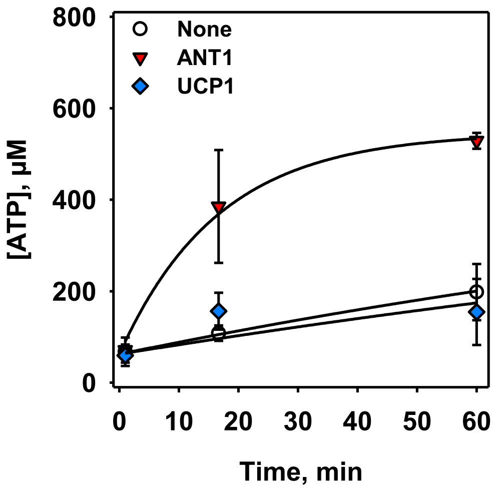

Supplementary Fig. 3 Time course of the increase of the ATP concentration inside (proteo-) liposomes measured with a ^{3}H-ATP uptake assay in the presence of ANT1 (triangles), UCP1 (diamonds) and in the absence of protein (circles). Lines are fits of an exponential rise to maximum function to the data. In the measurements, buffer contained 50 mM Na_2SO_4, 10 mM Tris, 10 mM MES and 0.6 mM EGTA at pH = 7.34 and T = 297 K. Lipid membranes were made of 45:45:10 mol% of DOPC, DOPE, and CL, respectively. The lipid concentration was 1.0 mg/mL and liposome diameter was approximately 100 nm. The concentrations of ANT1 and UCP1 were 8.67 µg/mL and 7.07 µg/mL, respectively, and the concentrations of ATP and ADP were 2 mM each. Data are shown as the mean ± SD of at least three independent measurements.
